# Automatic detection and characterization of quantitative phase images of thalassemic red blood cells using a mask region-based convolutional neural network

**DOI:** 10.1117/1.JBO.25.11.116502

**Published:** 2020-11-13

**Authors:** Yang-Hsien Lin, Ken Y.-K. Liao, Kung-Bin Sung

**Affiliations:** a National Taiwan University, Graduate Institute of Biomedical Electronics and Bioinformatics, Taipei, Taiwan; b Feng Chia University, College of Information and Electrical Engineering, Taichung, Taiwan; c National Taiwan University, Department of Electrical Engineering, Taipei, Taiwan; d National Taiwan University, Molecular Imaging Center, Taipei, Taiwan

**Keywords:** digital holographic microscopy, quantitative phase imaging, red blood cell, thalassemia, mask region-based convolutional neural network

## Abstract

**Significance:** Label-free quantitative phase imaging is a promising technique for the automatic detection of abnormal red blood cells (RBCs) in real time. Although deep-learning techniques can accurately detect abnormal RBCs from quantitative phase images efficiently, their applications in diagnostic testing are limited by the lack of transparency. More interpretable results such as morphological and biochemical characteristics of individual RBCs are highly desirable.

**Aim:** An end-to-end deep-learning model was developed to efficiently discriminate thalassemic RBCs (tRBCs) from healthy RBCs (hRBCs) in quantitative phase images and segment RBCs for single-cell characterization.

**Approach:** Two-dimensional quantitative phase images of hRBCs and tRBCs were acquired using digital holographic microscopy. A mask region-based convolutional neural network (Mask R-CNN) model was trained to discriminate tRBCs and segment individual RBCs. Characterization of tRBCs was achieved utilizing SHapley Additive exPlanation analysis and canonical correlation analysis on automatically segmented RBC phase images.

**Results:** The implemented model achieved 97.8% accuracy in detecting tRBCs. Phase-shift statistics showed the highest influence on the correct classification of tRBCs. Associations between the phase-shift features and three-dimensional morphological features were revealed.

**Conclusions:** The implemented Mask R-CNN model accurately identified tRBCs and segmented RBCs to provide single-RBC characterization, which has the potential to aid clinical decision-making.

## 1 Introduction

Information about red blood cell (RBC) morphology is crucial to reach a diagnosis of blood disorders because RBC morphologies often change due to altered membrane lipid composition, iron deficiency, or metabolic abnormalities.^1^^,^^2^ The current diagnostic procedure for many blood-related diseases is the blood smear test in which the morphology of stained RBCs is examined under a light microscope. Staining of the blood film is usually required to achieve adequate image contrast for proper microscopic examination. However, the staining process is time-consuming and may cause variations in the resulting contrast, both of which impede the blood smear test to be used as an automatic and high-throughput procedure.^3^

A promising label-free microscopy technique for high-throughput examination of blood smears is quantitative phase imaging (QPI).^4^^–^^6^ QPI is capable of mapping in two dimensions the phase shift caused by cellular constituents when a source light beam transmits through a transparent cell. The measured phase shift represents the line integral of the refractive index (RI) contrast between the specimen and its environment along the light path, which is parallel to the optical axis and corresponds to the physical thickness of the specimen. Since RBCs are mostly composed of hemoglobin and are without many organelles, the RI within an RBC is approximately constant and proportional to its hemoglobin concentration.^7^ Therefore, the phase shift of each pixel in an RBC phase image combines information about the hemoglobin concentration and thickness of the RBC at that position. Furthermore, integrating the phase shift over all pixels of an RBC phase image generates the so-called optical volume, which is proportional to the total dry mass of hemoglobin^8^ in the RBC. The optical volume has been shown to be a major feature in identifying hypochromic RBCs, as seen in iron deficiency anemia^9^^,^^10^ and thalassemia.^10^^,^^11^ In addition, phase-shift statistics and two-dimensional (2-D) morphological features, such as the projected area, perimeter, and lengths of major and minor axes, have been extracted from quantitative phase images of RBCs and have been used to classify blood disorders, including hereditary spherocytosis,^4^^,^^9^ malaria,^12^^–^^14^ and sickle cell disease,^15^^–^^17^ where RBCs possess abnormal hemoglobin content and show abnormal shape. These advances highlight a major advantage of using QPI for the diagnosis of RBC-related diseases: simultaneously quantifying diagnostically relevant hemoglobin content and morphological information without exogenous labels.

To further develop QPI to become a practical tool for automatic detection of RBC-related diseases, the conventional image processing approach^18^^,^^19^ consists of multiple steps including the segmentation of individual RBCs from QPI data, extraction and selection of features, and training of a classifier. For the automatic segmentation of multiple RBCs from a single quantitative phase image, Yi et al.^20^ have proposed a method based on marker-controlled watershed transform algorithm. This method addressed the issues of overestimation and underestimation in simple thresholding such as the Otsu method and is less susceptible to noise. However, the proposed algorithm is time-consuming and hence not suitable for real-time processing in clinical applications. As for selecting an optimal set of features to achieve accurate classification, special domain knowledge is required, and this selection is specific for each disease since different diseases present distinctive characteristics. The efficiency of both the development of an automatic tool and the inference of the results by the tool is not optimal using the conventional image processing approach.

The efficiency issues can be addressed by emerging deep-learning techniques, such as convolutional neural networks (CNNs).^21^^,^^22^ In addition to fast inference without time-consuming image processing, CNNs greatly simplify the development of image-based automatic diagnostic tools since feature extraction and selection are automatic and do not require any domain knowledge. Various CNN models have been applied to quantitative phase images to accurately classify 19 bacterial species,^23^ distinguish anthrax spores from five *Bacillus* species for biodefense,^24^ and detect breast cancer cells in whole blood samples for early cancer diagnosis and treatment response assessment.^25^ Other CNN models have been developed to segment healthy RBCs (hRCBs) in quantitative phase images^26^ and to detect RBCs with sickle cell disease^27^^–^^29^ or RBCs infected with malaria^30^ based on stained blood smear images. However, no CNN model has been established to segment and detect thalassemic RBCs (tRBCs) in quantitative phase images. Additionally, the black-box nature of deep-learning techniques has limited the interpretation of results, which has impeded the adoption of CNN models in medicine. In diagnostics, a comprehensive analysis of single cells is highly desirable to provide detailed morphological and chemical characteristics, thus aiding both the diagnosis and treatment planning. Hence, single-cell segmentation is a critical step to obtain the regions of interest (ROI) and to subsequently characterize single cells. An efficient deep-learning technique, mask region-based CNN (Mask R-CNN), has been proposed to automatically achieve instance segmentation of every recognized object.^31^ Since Mask R-CNN can achieve detection and segmentation simultaneously, it has been applied to automatically analyze fluorescence images of immune cells^32^ and to detect and segment nuclei in images of hematoxylin and eosin-stained histopathology slides of multiple organs^33^^,^^34^ and cancer tissues.^33^^,^^35^

In this paper, we demonstrate the implementation of Mask R-CNN for the automatic discrimination between quantitative phase images of hRBCs and those of tRBCs. Thalassemia is an inherited anemia that has a high prevalence (3.6%)^36^ worldwide. Owing to the early onset and detrimental effects of thalassemia, a minimally invasive diagnostic method for infants is needed to predict the disease severity and, then, to adopt timely and suitable treatments. QPI is well suited for this application because it requires only a drop of blood sample. In the current study, single-shot digital holographic microscopy (DHM) was used to acquire 2-D quantitative phase images of RBCs collected from thalassemia-minor patients and healthy subjects. A Mask R-CNN model was then trained to perform end-to-end detection of tRBCs. The classification accuracy of the model was benchmarked against an optimal classifier, which was built with the XGBoost technique and trained with 15 single-cell features, including phase-shift statistics, 2-D morphological features, and textural features extracted from manually segmented RBC phase images. To demonstrate the advantage of the Mask R-CNN model that provides instance segmentation, we extracted single-cell features from automatically segmented RBC phase images and trained a second XGBoost classifier to discriminate tRBCs from healthy ones. The features that contributed the most to successfully distinguish tRBCs were obtained using the SHapley Additive exPlanation (SHAP) analysis,^37^^,^^38^ which is based on game theory. Moreover, we analyzed correlations between the 2-D QPI features and three-dimensional (3-D) morphological features obtained with optical diffraction tomography of the same RBCs. Our findings suggest the use of QPI as a useful tool to capture 3-D morphological characteristics of abnormal RBCs, such as those seen in patients with thalassemia.

## 2 QPI Instrumentation and Data Acquisition

**Fig. 1:**
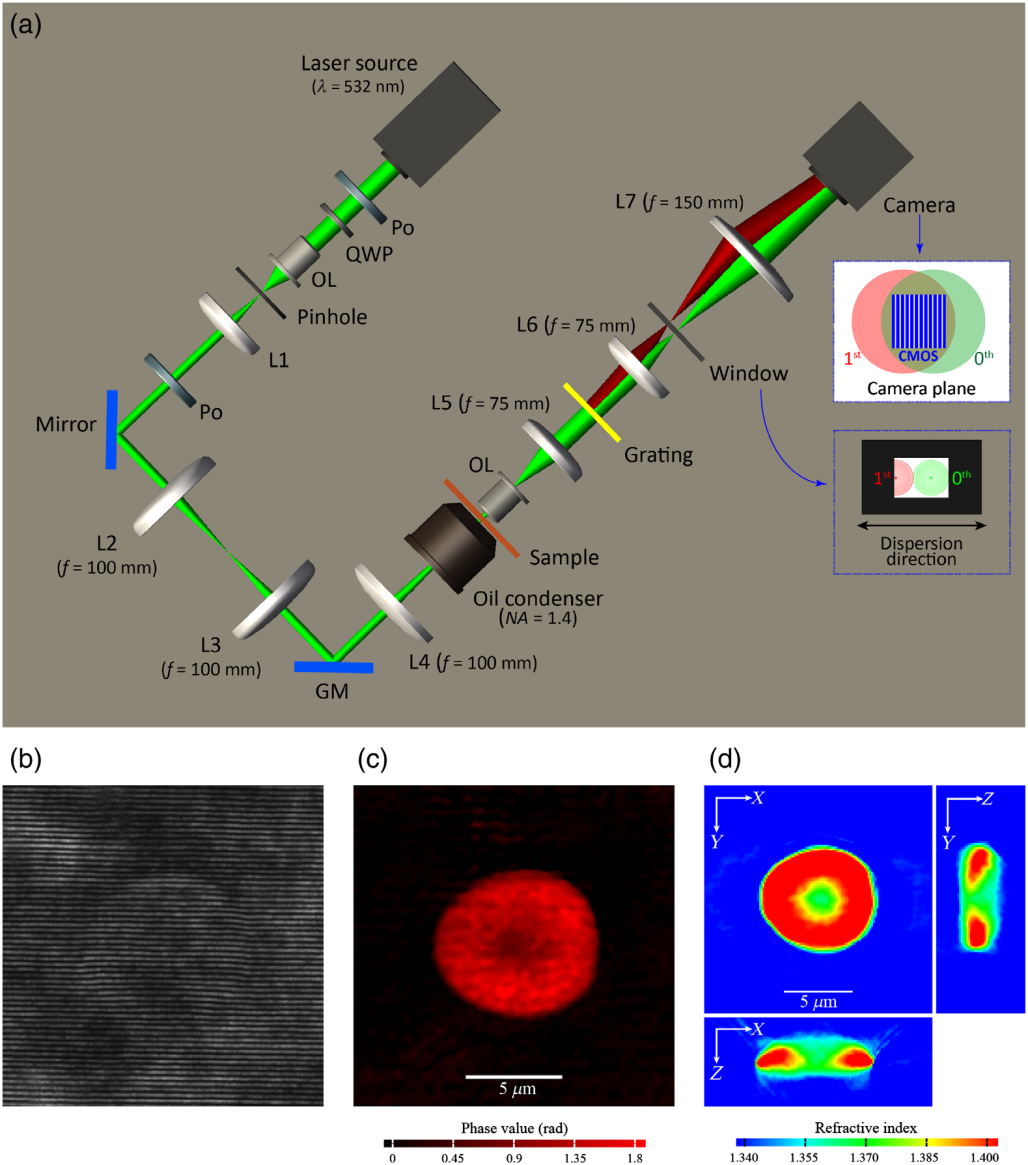
Schematic diagram of the DHM and imaging results of a hRBC. (a) Optical setup; upper inset: the red and the green areas depict the field of view of the first-order and the zeroth-order beam, respectively. Lower inset: the spatial frequency extent of the first-order and the zeroth-order beam is shown in red and green, respectively; dots in the center indicate the DC component. An interferogram of an hRBC is shown in (b), and the corresponding phase image and cross-sectional views of the RI tomogram are shown in (c) and (d), respectively. Po, polarizer; QWP, quarter-wave plate; OL1 and OL2, objective lens; L1 to L7, lenses; GM, galvanometer mirror.

We acquired 2-D quantitative phase images of RBCs using off-axis DHM. Details of the optical setup are described in Ref. 39 and a schematic diagram is shown in Fig. 1(a). A laser beam from a 532-nm continuous-wave laser goes through an oil-immersion condenser to generate a planar wavefront, illuminating the specimen at normal incidence. An objective lens (Olympus UPLSAPO 100XO, 1.4 NA) collects the transmitted field to project a magnified image of the specimen on a CMOS camera sensor (GZL-CL-41C6M-C, Gazelle, Point Grey). A transmission grating (Edmund, 80  grooves/mm) located after the tube lens (L5) generates multiple diffracted beams to provide a uniform reference beam that goes through the same components as the sample beam, before interfering with the sample beam at the camera sensor. A window is placed at the Fourier plane of L6 to allow the full zeroth-order beam and the DC signal of the first-order beam to pass. As shown in the inset of Fig. 1(a), the field of view in the zeroth-order beam is shifted from that in the first-order beam by moving the grating slightly off the rear focal plane of L5. At the camera sensor plane, the image of the specimen in the zeroth-order beam (henceforth the sample beam) overlaps with an empty area in the first-order beam (henceforth the reference beam) to form interference images of the specimen [as shown in Fig. 1(b)], which are recorded with a transverse magnification of approximately ×85. The same optical setup has been used to acquire quantitative phase images of RBCs under various incident angles by rotating a galvanometer mirror (GM, Cambridge Technology Inc.) located at a conjugate plane of the specimen.^11^ 3-D RI maps have been reconstructed based on optical diffraction tomography with direct interpolation in the Fourier domain and the positivity constraint.^40^ An example of a reconstructed phase image and RI map of an hRBC are demonstrated in Figs. 1(c) and 1(d).

This study was approved by the Institutional Review Board of National Taiwan University Hospital, and an informed consent was obtained from each subject. We imaged 210 hRBCs from 11 healthy subjects and 475 tRBCs from 29 adult thalassemia-minor patients. About 10 interference images were acquired from each RBC to expand the dataset. In total, 2001 hRBCs and 4268 tRBCs interference images were collected. Phase images were retrieved from the interference images by bandpass filtering in the spatial frequency domain and inverse Fourier transform without zero-padding,^41^ followed by a discrete cosine transform-based phase unwrapping method.^42^ We implemented the phase retrieval and unwrapping processes by parallel computing on a NVIDIA GTX 1080 Ti GPU and an Intel Core i7-3820 CPU, enhancing the computation speed to about 289 fps.

Since the number of tRBC images was about two times more than that of hRBC images, we adopted data augmentation by random rotation, to enlarge the hRBC dataset to balance data for XGBoost classifier training and Mask R-CNN model building. Furthermore, the operation of random rotation increased the variation in datasets, to prevent overfitting during Mask R-CNN model training.^43^^–^^45^ The final total number of hRBC images was equal to that of tRBC.

## 3 Automatic Detection and Delineation of RBCs

### 3.1 Development of the Mask R-CNN Model

**Fig. 2:**
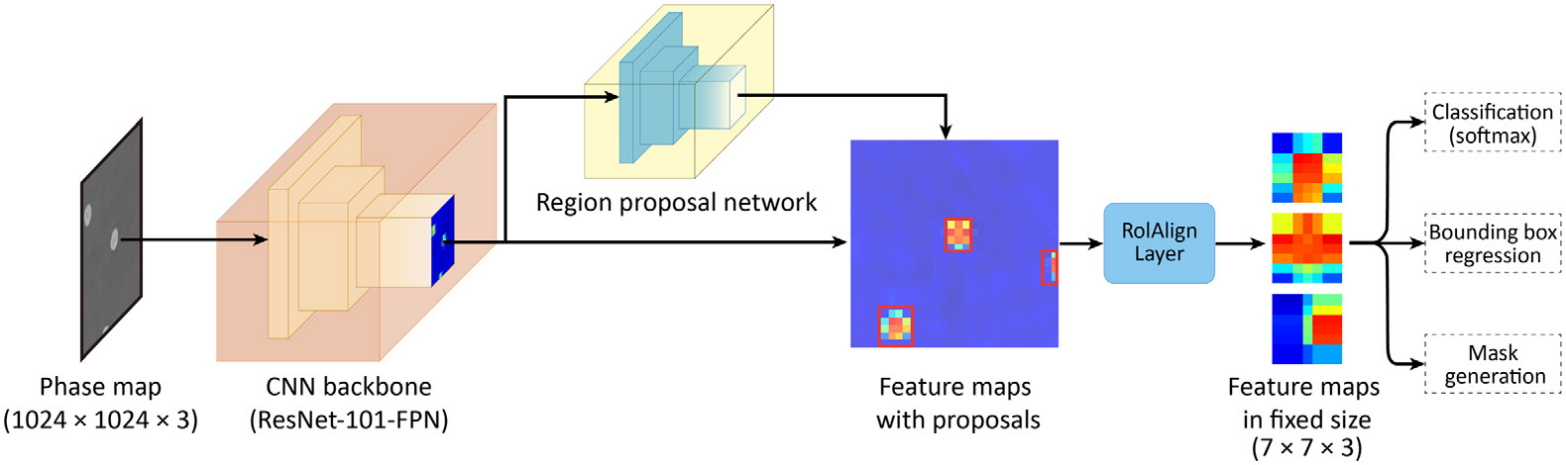
Illustration of Mask R-CNN architecture for instance segmentation and classification. A CNN extracts image features, and a region proposal network generates ROIs containing individual RBCs. The RoIAlign layer scales every ROI to the same size and passes the ROIs to each branch to perform RBC detection and segmentation.

The Mask R-CNN model was developed using the Deep-Learning-Framework Keras (v2.3.1) and TensorFlow (v1.12.0) as the backend, and accelerated using NVIDIA CUDA (v10.0) and cuDNN (v7.5). The architecture of the Mask R-CNN model, shown in Fig. 2, can be split into two stages. Tasks of the first stage were image scanning and proposal generation. The tasks of the second stage were object classification,^46^ bounding box coordinates generation,^46^ and mask generation^31^ for each RBC in a quantitative phase image. The two stages were combined into a single workflow that can be trained end-to-end, achieving pipeline optimization and optimal performance.^31^ A 101-layer CNN backbone with a feature pyramid network, ResNet-101-FPN,^46^ was used to extract high-level image features via back propagation. To feed the phase images into the backbone to obtain feature maps, single-channel phase maps were replicated into three-channel images (1024×1024×3 channels). A region proposal network was then applied to predict candidate bounding boxes of objects (RBCs) from the high-level image feature maps. An RoIAlign layer scaled every candidate bounding box to the same size (7×7×3 channels) using bilinear interpolation to retain floating-point numbers for subsequent tasks. The scaled ROI feature maps were then passed to fully connected layers to infer the object class and to determine bounding box offset values. In parallel, masks (28×28×3 classes) were generated from the scaled ROI feature maps in the mask branch with multiple convolutional layers.

We partitioned the augmented dataset described in Sec. 2 into training, validation, and test datasets with a ratio of 3:1:1. The initial learning rate, batch size, and steps per epoch were 0.002, 64, and 80, respectively. The categorical cross-entropy of classification and the intersection over union (IoU)^47^ of masks were calculated on the validation dataset to monitor the performance of classification and segmentation, respectively.

### 3.2 Development of the Benchmark Classifier Using XGBoost

To benchmark the performance of the proposed Mask R-CNN model in distinguishing tRBCs from hRBCs, we built an optimal classifier with features extracted from manually segmented RBC quantitative phase images. Table 1 lists the 15 features that were used. The optical and morphological features were readily obtained from each segmented RBC phase image, and the textural features were calculated using the gray-level co-occurrence matrix (GLCM)^48^^,^^49^ of each segmented RBC image to quantify its spatial hemoglobin distribution. GLCM is a statistical method to describe the textural structure of an image by statistically sampling the pattern of gray-level occurrences in relation to other gray levels. The benchmark classifier was built using XGBoost (eXtreme Gradient Boosting), an open-source library of the gradient boosted trees algorithm.^50^ XGBoost is a supervised machine learning technique that combines a set of classification and regression trees to predict a target variable. XGBoost uses a regularized objective function to evaluate the classification accuracy and simplification of the ensemble model and decide whether a new tree is to be ensembled to the prior model or not. XGBoost has been widely used for classification tasks and has won many machine learning competitions in Kaggle, due to its advantages in flexible model tuning, accelerated computing, and enhancements of various algorithm.^51^ The hRBC dataset was expanded as described in Sec. 2, and the augmented dataset was separated into a training dataset and a test dataset in a ratio of 4:1 for the XGBoost classifier.

**Table 1:** List of features used in the XGBoost classifier.

Category	Features
Phase-shift statistics (n=5)	Optical volume, mean, fifth percentile, 95th percentile, and standard deviation of phase shift
Morphological property (n=5)	Projected area, perimeter, major axis length, minor axis length, and eccentricity
Textural property based on the GLCM (n=5)	Contrast, dissimilarity, homogeneity, energy, and angular second moment^49^

### 3.3 Results of RBCs Detection and Delineation

Table 2 summarizes the classification performance of Mask R-CNN model and the benchmark XGBoost classifier on the test dataset. The Mask R-CNN model achieved high accuracy (97.8%) in the test dataset, which is very close to the accuracy obtained by the benchmark XGBoost classifier (accuracy 99.9%).

**Table 2:** Comparison of classification performance on individual RBCs using Mask R-CNN, XGBoost with manual delineation and XGBoost with Mask R-CNN segmentation results.

Method	Sensitivity (%)	Specificity (%)	F1-score	AUC	Accuracy (%)
Mask R-CNN	97.1	98.5	0.975	0.982	97.8
XGBoost with MD	99.9	100	0.992	0.999	99.9
XGBoost with Mask R-CNN^a^	99.9	100	0.992	0.999	99.9

Note: AUC, area under the receiver operating characteristic curve; MD, manual delineation.

a XGBoost classifier trained with the segmentation results from Mask R-CNN.

**Fig. 3:**
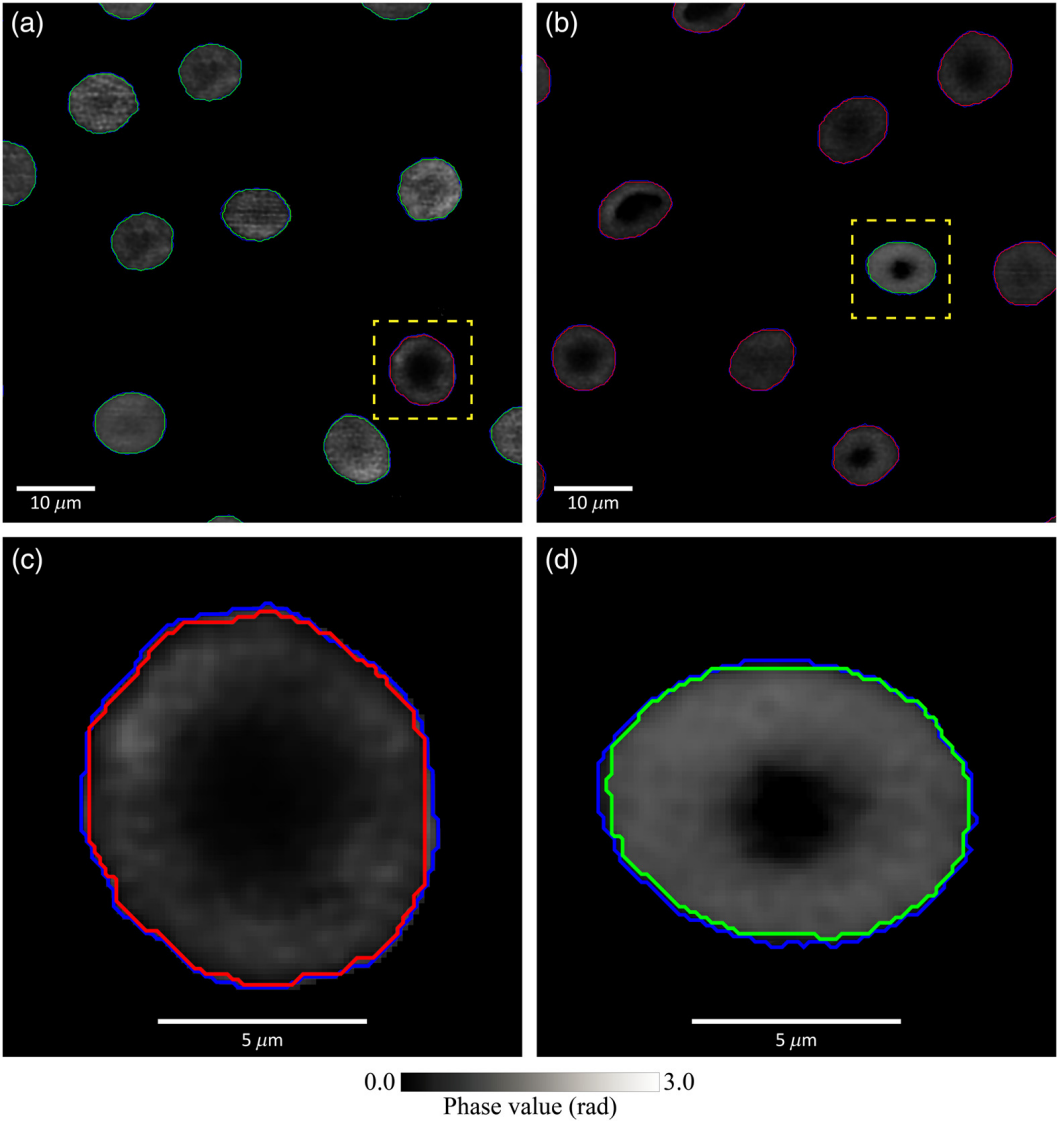
Exemplary results of the delineation and detection by the Mask R-CNN model. Two slides with hRBCs and tRBCs are shown in (a) and (b). Blue lines are the manual delineation contours of RBCs as the ground truth. Green and red lines mark mode-predicted contours of RBCs classified as hRBCs and tRBCs, respectively. The misclassified cases in (a) and (b) are enlarged and shown in (c) and (d). The model-predicted contours match well with manual delineation contours, indicating that the misclassification is not due to failed segmentation.

Exemplary results of segmenting RBCs with the Mask R-CNN model are illustrated in Fig. 3, and corresponding ground truths by manual delineation are shown in blue lines in Fig. 3 for comparison. The segmentation performance of the Mask R-CNN model was evaluated using IoU between the model-predicted mask and the ground-truth mask. The average IoU of the test dataset was 0.945. The RBC phase images, segmented by the Mask R-CNN model, were utilized to train a second XGBoost classifier using the procedure described in Sec. 3.2. The classification performance, also listed in Table 2, was the same as that obtained by the benchmark XGBoost classifier trained with manually delineated RBC images. These results show that the Mask R-CNN model accurately segmented RBC quantitative phase images.

## 4 Characterization of Thalassemic RBC Quantitative Phase Images

**Fig. 4:**
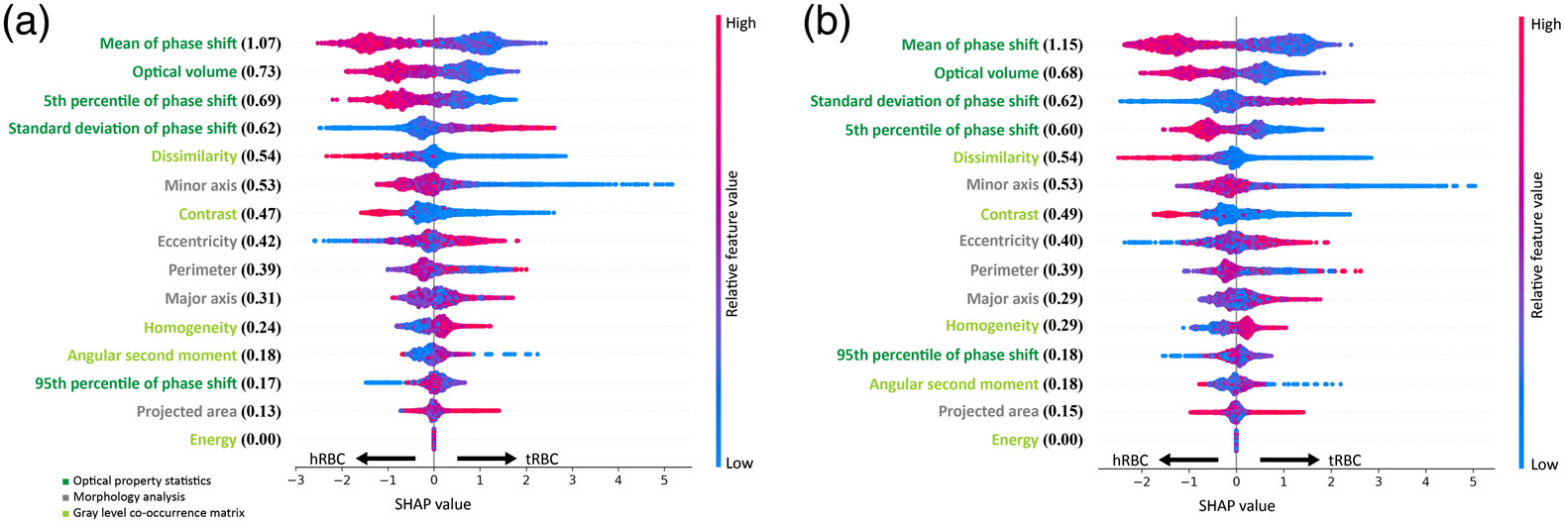
SHAP value distributions of the 15 selected features for XGBoost classifier trained with (a) Mask R-CNN segmentation and (b) manual segmentation. The features are sorted according to the mean absolute SHAP value that represents the relative contribution of each feature to the XGBoost classifier. The color presents the normalized feature value of each RBC in the test dataset.

To unveil the underlying characteristics of RBCs that enable the accurate classification of tRBCs and to demonstrate an advantage of Mask R-CNN’s in performing instance segmentation, we took the segmentation results of the Mask R-CNN model to train a second XGBoost classifier for discriminating tRBCs from hRBCs. Then, we performed the SHAP analysis to evaluate the relative impact of each feature on the XGBoost classifier.^52^^–^^55^ Each dot in Fig. 4 indicates the SHAP value of an RBC in the test dataset for the feature mentioned on the left. The color of a dot indicates the measured value for the corresponding feature, with higher values in red and lower values in blue. The magnitude of the SHAP value indicates the impact level of each data point to the model. Therefore, the overall impact of each feature can be quantified by the sum of the absolute SHAP values, over all the data points. In Fig. 4, the distributions of relative feature values also indicate the feature representations of each class. For example, tRBCs demonstrated a low optical volume, mean, and fifth percentile of phase shift but a high standard deviation of phase shift. The most influential features, with a mean absolute SHAP value larger than 0.6, are all related to phase shifts, including the mean, the sum (optical volume), the fifth percentile, and the standard deviation of phase shifts, of single RBCs. On an average, the morphological features showed a moderate influence and the textural features showed the least influence, on the XGBoost classifier. The lack of significant contributions from the textural features could be attributed to approximately uniform hemoglobin distributions in both hRBCs and tRBCs. However, in patients with severe anemia or malaria, where RBCs are nucleated or infected, the texture analysis may provide important information for detecting the abnormal RBCs. SHAP analysis was also performed on the benchmark XGBoost classifier. The result is shown in Fig. 4(b) and is very similar to that of the XGBoost classifier trained with Mask R-CNN segmented RBC images shown in Fig. 4(a). These results demonstrated that there were no significant differences in the discriminant rules between the classifier trained with the Mask R-CNN segmentation and the classifier trained with the manual delineation.

## 5 Correlations between the Features Extracted from 2-D Quantitative Phase Images and 3-D RI Maps of RBCs

Results shown in Table 2 indicate that 2-D QPI-extracted features are sufficient to accurately discriminate tRBCs from hRBCs. According to the SHAP analysis described in Sec. 4, the most influential features are related to the phase shifts of RBCs. Since the phase shift is the line integral of the RI contrast along the RBC thickness, it is plausible that the 2-D QPI features are correlated to the 3-D morphological information for distinguishing tRBCs from hRBCs. To elucidate correlations between the phase-shift statistics and features extracted from 3-D RI maps of the same RBCs, we applied the canonical correlations analysis (CCA) to investigate associations between the two sets of features.

### 5.1 Procedure of the Canonical Correlation Analysis

CCA is a statistical method for exploring correlations between two sets of variables acquired on the same experimental units.^56^ Assume X and Y denote two sets of continuous variables. CCA aims to seek two vectors a and b, canonical coefficients, to maximize the correlation between linear combinations of X and Y formulated as $U=a^{T}X$ and $W=b^{T}Y$. The pair of vectors U and W is called canonical variates, and the correlation between U and W is quantified to represent the canonical correlation. After the first pair of canonical variates is obtained, a new pair of canonical variates can be found by maximizing their correlation under the condition that they are uncorrelated with previously extracted canonical variates. The process of canonical variate extraction repeats until the number of canonical variates equals to the minimum feature number between X and Y.

The top four 2-D QPI features identified by the SHAP analysis (Sec. 4) included optical volume (OV), mean ($\phi_{\text{mean}}$), fifth percentile ($\phi_{P5}$), and the standard deviation ($\phi_{\mathrm{SD}}$) of single-RBC phase shifts and were chosen as predictor variables (X). For selecting features of the 3-D RI maps, we chose features that demonstrated relatively good performance in discriminating tRBCs from hRBCs (the area under the receiver operating characteristic curve, AUC>0.8), as reported in Table 3 of Ref. 11. These selected 3-D features were assigned as response variables (Y) and included the volume (V), surface-area-to-volume ratio (S/V), sphericity index $\left[SI=\pi^{1/3}{\left(6V\right)}^{2/3}/S\right]$, average thickness ($T_{\mathrm{avg}}$), central thickness ($T_{0}$), and the difference between the thickness at three quarters of the normalized radial position and $T_{0}$ ($\Delta_{75}$). Each of the variables was standardized by removing the mean and scaling to unit variance before CCA was performed. Since the number of predictor variables and response variables are p=4 and q=6, respectively, there are four pairs of canonical variates [min(p,q)=4] denoted as $\left(U_{i},W_{i}\right)$ with i=1, 2, 3, and 4.

Since the canonical correlation is calculated between a pair of canonical variates that are linear combinations of the original variables, it is desirable to analyze associations between the canonical variates and the original variables. We followed the method described by Arifler^57^ that used within-set loadings to assess the associations. For example, a predictor variable is interpreted to be highly correlated with a canonical variate $U_{i}$ if it has a relatively high canonical loading. Similarly, the canonical loadings of response variables can be used to interpret their correlations with $W_{i}$.

### 5.2 Associations between Features Extracted from 2-D Quantitative Phase Images and 3-D RI Maps of the Same RBCs

The canonical correlations (ρ) and canonical loadings (L) of CCA are listed in Table 3. The top two canonical variate pairs showed very high correlations (ρ=0.94 and 0.87, respectively). The third and fourth pairs of canonical variates showed weak correlations (ρ=0.27 and 0.15, respectively) and will not be discussed further. The first pair of canonical variates, $U_{1}$ and $W_{1}$, is dominated by OV and V, respectively. Since phase shift is the product of RI and RBC thickness, and OV is the sum of the phase shift over the whole RBC image, OV is expected to be proportional to RBC volume if the average RI does not vary significantly between RBCs from the same subject.^58^ Both OV and V concern the volume of an RBC, not the shape of it. In contrast, an inspection of canonical loadings of the second canonical variate pair, $U_{2}$ and $W_{2}$, indicates associations between the other phase-shift statistics and various shape-related features. Following the same assumption that there is no significant difference in the average RI between RBCs from the same subject, the phase shift is proportional to the thickness of an RBC. Therefore, $\phi_{\text{mean}}$ is proportional to $T_{\mathrm{avg}}$ and $\phi_{P5}$ and is closely related to $T_{0}$. Figures 3 and 5 of Ref. 11 show that tRBCs tend to be shaped like doughnuts, which is associated with increase in $\phi_{\mathrm{SD}}$, S/V, and $\Delta_{75}$, and with a decrease in SI. The canonical loadings of $U_{2}$ and $W_{2}$ also indicate that $\phi_{\mathrm{SD}}$ is positively correlated with S/V and $\Delta_{75}$ and negatively correlated with SI. The results of CCA reveal associations between the phase-shift statistics and the 3-D morphological features of RBCs and facilitate the interpretation of 2-D QPI-based features that achieved excellent accuracy in distinguishing tRBCs from hRBCs.

**Table 3:** CCA between phase-shift statistics extracted from 2-D quantitative phase images and features extracted from 3-D RI maps.

Pairs of canonical variates	First	Second	Third	Fourth
Canonical correlations (ρ)	0.94	0.87	0.27	0.15
Shared variances ($\rho^{2}$)	0.89	0.76	0.07	0.02
	Canonical loadings			
	$U_{1}$	$U_{2}$	$U_{3}$	$U_{3}$
OV	−0.53	−0.51	0.14	−0.04
$\phi_{\text{mean}}$	0.13	−0.60	0.17	−0.06
$\phi_{\mathrm{SD}}$	0.27	**0.53**	0.19	0.09
$\phi_{P5}$	−0.10	−0.69	0.05	−0.04
	$W_{1}$	$W_{2}$	$W_{3}$	$W_{4}$
V	−0.67	−0.55	0.06	0.00
S/V	0.26	**0.77**	−0.06	0.03
SI	0.18	−0.79	0.10	−0.02
$T_{\mathrm{avg}}$	−0.06	−0.81	0.06	0.03
$T_{0}$	−0.04	−0.74	0.04	0.06
$\Delta_{75}$	−0.00	**0.81**	0.02	−0.05

Note: Bold values show the highest correlation in each canonical loading.

## 6 Discussion and Conclusions

We successfully applied Mask R-CNN to build a classification model to accurately (accuracy=97.8%) discriminate between hRCBs and tRBCs, based on quantitative phase images of unstained RBCs. In the development phase of the Mask R-CNN model, a CNN backbone was adopted to automatically extract deep features and to replace the manual feature extraction and selection steps employed in conventional image processing-based automatic diagnostic methods. Therefore, the requirement for hematological knowledge to select optimal features is eliminated. During inference, the Mask R-CNN model can efficiently and automatically detect tRBCs. In addition, the same Mask R-CNN model also provided segmentation of RBCs for single-cell characterization of 2-D QPI features. The results shown in Sec. 4 reveal the most important 2-D QPI features of tRBCs that can separate them from hRBCs. In future clinical applications, these features can be quantified automatically and used by healthcare providers to aid their decision-making processes.

Figure 4 shows that the most influential features for the classification of tRBCs are related to phase shifts. The optical volume (i.e., the sum of phase shifts) of an RBC is proportional to the dry mass of the RBC. It is worth to note that the mean corpuscular hemoglobin obtained using a regular blood test (i.e., complete blood count) is the average mass of hemoglobin per RBC in the whole blood sample. Therefore, QPI of RBCs provides not only the average hemoglobin content per RBC but also the hemoglobin content of every RBC imaged. Results of the CCA analysis show that the other phase-shift statistics are highly associated with various 3-D morphological features of RBCs (see Table 3 in Sec. 5.2). To the best of our knowledge, correlations between phase shifts and the 3-D morphology of tRBCs have not been experimentally investigated and reported. Mugnano et al.^10^ characterized the phase distribution of individual RBCs by fitting quantitative phase images of RBCs with Zernike polynomials. Three Zernike coefficients, namely, the piston, the defocus, and the third-order spherical aberrations, were identified to best distinguish tRBCs from hRBCs. The piston is related to the size and the mean phase shift of an RBC, and so is the optical volume. The defocus and the third-order spherical aberration are associated with the biconcave shape of RBCs. The 3-D morphological features analyzed in the current study are more intuitive and commonly used, and thus allow for direct comparisons with existing studies in the literature.^59^^–^^64^

To realize high-throughput screening of RBCs for clinical applications, both the hardware and the software reported in this paper need to be improved. First, the QPI instrument can be integrated with microfluidic devices to automate the dilution and pumping of blood samples for optimal acquisition speed with sufficient image quality.^5^^,^^65^ Second, rapid processing and inference of quantitative phase images using the Mask R-CNN method is needed for real-time RBC detection and delineation. Currently, the inference time of the Mask R-CNN model on a phase map with one million pixels is under 0.2 s using an NVIDIA GTX 1080 Ti GPU. To improve the speed of the inference, the CNN backbone can be further optimized by combining layers and optimizing the kernel selection, for instance, using inference accelerators such as the TensorRT.^66^ We believe that the inference frame rate of the proposed Mask R-CNN model can achieve video rate on images with one million pixels, using our current computing hardware. Furthermore, due to its excellent automatic detection and high deplorability, the proposed Mask R-CNN model is well suited for assisting diagnostics via telehealth in remote areas lacking medical resources.

In conclusion, we demonstrated simultaneously the automatic delineation of RBC phase images for single-cell analysis and highly accurate detection of tRBCs using Mask R-CNN. The developed Mask R-CNN model efficiently processed massive amounts of QPI data and greatly simplified the process of developing an automatic method for detecting RBC-related abnormalities from the QPI data. Moreover, the instance segmentation capability of the Mask R-CNN model was very useful for single-cell characterization, and combined with a classifier and the SHAP analysis, provided invaluable insights into the relationship between RBC QPI features and diseases. To help interpret the QPI features, CCA was performed to elucidate associations between the statistics of the phase shift and the more intuitive RBC 3-D morphology. We believe that Mask R-CNN has the potential to improve the efficiency of hematological examinations and achieve single-RBC characterization to aid clinical decision-making.
